# Supplementary material for: Simple and Efficient Pressure Ulcer Reconstruction via Primary Closure Combined with Closed-Incision Negative Pressure Wound Therapy (CiNPWT)—Experience of a Single Surgeon
Source: J Pers Med. 2022 Jan 29;12(2):182. doi: 10.3390/jpm12020182 (PMC8875003; doi:10.3390/jpm12020182)
Supplement: Supplementary file 1 [file jpm-12-00182-s001.zip › Pressure Ulcer - Supplementory data 21220207.pdf]

Patient	Wound	Sex	Age	Type of defect	Cause of defect	Co-morbidities	Wound culture	Debridement (times)	Flap type	Follow months	Operation time	wound size (cm2)	Hospital stay (day)	Healed	Minor complication	Major complication	Mortality
1	1	F	96	Sacral	Spinal cord compression, T11/12 level due to disk bulging and corresponding flaval ligament hypertrophy with monoparesis, left lower leg	HTN, PAOD, CKD, Hyperlipidemia, Depressive disorder, Hyperuricemia	*. Pseudo. aeruginosa<br>*. Enterococcus faecalis<br>*. Bacteroides fragilis	2	PC+CiNPWT	22	36	35	21	1	0	0	0
2	2	F	90	Sacral	Dementia	DM2, SSS, Hx of pacemaker implantation, Hyperlipidemia, Major depression disorder	*. Staphylococcus aureus<br>*. Proteus mirabilis<br>*. Escherichia coli<br>*. Bacteroides fragilis	2	PC+CiNPWT	14	41	70	30	1	0	0	0
3	3	F	67	Sacral	Parkinson's disease	nil	*. Proteus mirabilis	2	PC+CiNPWT	22	49	63	31	1	0	0	0
4	4	F	84	Sacral	Old CVA	HTN, DM2	*. Morganella morganii<br>*. Escherichia coli<br>*. Veillonella spp.<br>*. Bacteroides fragilis<br>*. Fusobacterium nucleatum	2	PC+CiNPWT	3	46	130	55	0	1	0	0
5	5	F	84	Sacral	Old CVA	HTN, DM2	0	1	PC+CiNPWT	12	42	63	26	1	0	0	0
6	6	F	91	Sacral	Fracture of intertrochanter of femur	DM, HTN, Old CVA	*. Enterobacter cloacae<br>*. Enterococcus faecium<br>*. Bacteroides fragilis<br>*. Peptostrepto. Anaerobicus<br>*. Viridans streptococus gr.	3	PC+CiNPWT	18	27	54	32	1	0	0	0
7	7	F	71	Right ischial	Spinal hemorrhage complicated spinal cord compression with paralysis s/p decompression	nil	*. Enterococcus faecalis<br>*. Peptostrepto. Magnus<br>*. Bacteroides fragilis<br>*. Bacteroides ovatus<br>*. Bact. thetaiotaomicron	2	PC+CiNPWT	16	34	24	19	1	0	0	0
8	8	F	79	Sacral	L4 compression fracture with spinal stenosis (Proved MRI of L-spine was performed on 109/03/17 in 松山), with bed ridden.	nil	*. Pseudo. Aeruginosa<br>*. Viridans streptococus gr.<br>*. Bacteroides fragilis<br>*. Bacteroides uniformis	2	PC+CiNPWT	3	39	40	35	1	0	0	0
9	9	M	76	Sacral	Old CVA	DM2, HTN, Hyperlipidemia	*. Klebsiella pneumoniae<br>*. Pseudo. Aeruginosa<br>*. Bacteroides vulgatus	4	PC+CiNPWT	20	52	110	61	0	1	0	0
10	10	M	63	Sacral	CNS tumor	HTN	*. Escherichia coli<br>*. Candida albicans<br>*. Bact. Thetaiotaomicron	2	PC+CiNPWT	13	31	48	30	1	0	0	0
11	11	M	85	Sacral	Alzheimer's disease	HTN, COPD	*. Escherichia coli	1	PC+CiNPWT	14	45	20	30	1	0	0	0

12	12	M	95	Sacral	Senile dementia	HTN	*. Escherichia coli<br>*. Pseudo. aeruginosa<br>*. Peptostrepto. asaccharolyticus<br>*. Acineto. baumannii<br>*. Staphylococcus aureus	2	VY	6	54	100	42	1	0	0	0
12	13	M	95	L't trochanteric	Senile dementia	HTN	*. Escherichia coli<br>*. Pseudo. aeruginosa<br>*. Peptostrepto. asaccharolyticus<br>*. Acineto. baumannii<br>*. Staphylococcus aureus	3	pALT	4.5	221	90	86	1	0	0	0
12	14	M	95	Right hip	Senile dementia	HTN	*. Escherichia coli<br>*. Pseudo. aeruginosa<br>*. Peptostrepto. asaccharolyticus<br>*. Acineto. baumannii<br>*. Staphylococcus aureus	5	PC+CiNPWT	3.25	81	32	86	1	0	0	0
13	15	F	73	Sacral	Parkinsonism	DM2	*. Staphylococcus aureus<br>*. Coagulase Negative Staphylococcus<br>*. Corynebacterium spp.<br>*. Bact. thetaiotaomicron<br>*. Peptostrepto. Asaccharolyticus<br>*. Peptostrepto. Magnus	1	PC+CiNPWT	18	31	63	46	1	0	0	0
14	16	F	79	Sacral	Senile dementia	CHF, ESRD, HTN, DM2, VHD	*. Candida albicans<br>*. Enterococcus faecium<br>*. Pseudo. aeruginosa<br>*. Acinetobacter nosocomialis<br>*. Enterobacter cloacae	4	PC+CiNPWT	18	26	80	63	0	1	0	0
15	17	M	73	Sacral	Major depressive disorder	HTN	*. Bacteroides fragilis	2	PC+CiNPWT	3	39	90	22	1	0	0	0
16	18	M	52	Sacral	Ruptured cerebral aneurysm	nil	*. Enterococcus faecalis<br>*. Pseudo. aeruginosa<br>*. Peptostrepto. Anaerobicus	2	PC+CiNPWT	12	37	102	23	1	0	0	0
17	19	M	87	Sacral	Alzheimer's disease	nil	*. Enterococcus faecium<br>*. Pseudo. aeruginosa<br>*. Candida albicans	2	PC+CiNPWT	13	44	70	50	0	1	0	0
18	20	M	90	Sacral	Hypoxic encephalopathy	CKD, COPD, Hypothyroidism	*. Enterococcus raffinosus<br>*. Bact. thetaiotaomicron	2	PC+CiNPWT	1	56	198	43	0	1	0	1
19	21	F	89	Sacral	Senile dementia		*. Streptococus anginosus group<br>*. Bacteroides fragilis<br>*. Bact. distasonis	2	PC+CiNPWT	9	41	48	22	1	0	0	0
20	22	M	26	Sacral	Highly suspected Bickerstaff's brainstem encephalitis, also involves spinal cord with completely paraplegia, hypoesthesia (sensory level at T7-8 dermatome), and motor axonal neuropathy variant type, complicated with seizure	nil	*. Pseudo. Aeruginosa<br>*. Klebsiella pneumoniae<br>*. Candida albicans<br>*. Diphtheroid	2	PC+CiNPWT	18	54	130	67	1	0	0	0
21	23	M	61	Sacral	Cerivcal spinal cord injury	nil	*. Pseudo. Aeruginosa<br>*. Enterococcus casseliflavus	2	PC+CiNPWT	5	36	24	138	0	1	0	0

22	24	M	86	Sacral	*. Hip Fx s/p ORIF	HTN	0	1	PC+CiNPWT	1	22	40	15	1	0	0	0
23	25	F	66	Right hip	ICH	nil	0	5	pALT	5.25	126	104	75	1	0	0	1
23	26	F	66	Sacral	ICH	nil	0	4	PC+CiNPWT	1.5	52	120	75	1	0	0	1
24	27	M	58	Sacral	Old CVA	HTN, DM2	0	3	PC+CiNPWT	1	57	117	20	1	0	0	0
25	28	F	69	Left hip	Hydrocephalus	HTN, DM2	*. Bacteroides fragilis<br>*. Peptostrepto. magnus	2	PC+CiNPWT	16	24	56	27	1	0	0	0
25	29	F	69	Right hip	Hydrocephalus	HTN, DM2	*. Escherichia coli<br>*. Peptostrepto. Magnus<br>*. Viridans streptococus gr.	1	PC+CiNPWT	1.5	33	20	17	1	0	0	0
26	30	F	94	Right hip	Senile dementia	Arrythmia, Hyperlipidemia, CKD, VHD	*. Staphylococcus aureus<br>*. Pseudo. aeruginosa	2	PC+CiNPWT	1	21	54	17	1	0	0	0
27	31	M	74	Left hip	Old CVA	DM, HTN.	*. Staphylococcus aureus	2	PC+CiNPWT	1	24	30	14	1	0	0	0
28	32	M	75	Right hip	Hip fx s/p THR	DM2	*. Staphylococcus aureus<br>*. Providence stuartii<br>*. Proteus mirabilis<br>*. B-Streptococcus Non-A,B,D<br>*. Bacteroides fragilis<br>*. Peptostrepto. anaerobicus	2	PC+CiNPWT	5	25	24	19	1	0	0	0
29	33	M	94	Sacral	Senile dementia	HTN, CAD, Sick sinus syndrome (arrythmia)	0	1	PC+CiNPWT	3	45	54	19	1	0	0	0
30	34	F	97	Right back	Alzhiemer disease	HTN	No growth	4	Primary closure	24	50	42	26	1	0	0	0
31	35	F	82	Sacral	Parkinsonism	HTN, DM2, Parkinsonism, HIVD, MDD	No growth	1	V-Y	24	110	42	17	1	0	0	0
32	36	M	91	Sacral	Stroke	CAD, DM, HTN	Pseudo. aeruginosa, Bacteroides fragilis	3	V-Y	1.5	95	66	67	1	0	0	1
32	37	M	91	L't trochanter	Stroke	CAD, DM, HTN	Pseudo. aeruginosa, Bacteroides fragilis		Hatchet flap	1	160	120	67	1	0	0	1
33	38	M	79	Sacral	Head injury with ICH	HTN, CAD, DM2	Viridans streptococus, Eubacterium lentum	2	V-Y	24	77	42	32	0	1	0	0
34	39	F	60	Sacral	Old CVA with right hemiplegia	Nil	Pseudo. aeruginosa	5	V-Y	24	63	48	44	1	0	0	0
34	40	F	60	R't trochanter	Old CVA with right hemiplegia	Nil	Pseudo. aeruginosa	3	Primary closure	15	31	60	44	0	1	0	0

35	41	F	83	Sacral	Compression fracture of the L1	DM	Mixed flora	2	V-Y	14	88	55	40	1	0	0	0
35	42	F	83	upper back	Compression fracture of the L1	DM	Mixed flora	2	Primary closure	14	35	50	40	1	0	0	0
35	43	F	83	L't chest wall	Compression fracture of the L1	DM	Mixed flora	1	Primary closure	14	45	36	40	1	0	0	0
36	44	F	88	Sacral	Stroke	DM, CAD, CHF, Hyperlipidemia, atrial fibrillation, Hyperuricemia	Escherichia coli, MRSA	2	Primary closure	17	42	9	93	1	0	0	0
37	45	F	83	Sacral	Stroke	HTN	Escherichia coli, Bact. Thetaiotaomicron	2	V-Y	19	63	40	30	1	0	0	0
38	46	F	84	R't trochanter	Dementia	HTN, DM	Klebsiella pneumoniae, Enterococcus faecalis	2	Primary closure	3.75	30	25	54	1	0	0	0
38	47	F	84	L't trochanter	Dementia	HTN, DM	Klebsiella pneumoniae, Enterococcus faecalis	2	Primary closure	3.5	26	30	54	1	0	0	0
38	48	F	84	Sacral	Dementia	HTN, DM	Klebsiella pneumoniae, Enterococcus faecalis	5	SGAP	3	85	135	54	1	0	0	0
39	49	F	65	L't trochanter	Stroke	DM	Staphylococcus aureus, Proteus mirabilis, Diphtheroid	1	TFL hatchet flap	29	96	42	31	1	0	0	0
40	50	M	54	R't trochanter	Traumatic spinal cord injury	HTN, Alcoholism, Liver cirrhosis	Mixed flora	3	TFL	60	235	132	33	1	0	0	0
40	51	M	59	Sacral	Traumatic spinal cord injury	HTN, Alcoholism, Liver cirrhosis	Proteus mirabilis, Bacteroides fragilis	2	V-Y	0.5	96	160	24	0	1	0	1
41	52	F	76	Sacral	Cardiac arrest s/p ACLS with hypoxia	HTN, DM	No growth	1	SGAP	2.25	120	81	13	1	0	0	0
42	53	M	83	Sacral	CAD s/p CABG	CAD, Atrial fibrillation, DM, HTN, ESRD, Hyperuricemia.	Bacteroides fragilis	2	V-Y	4	70	90	114	0	0	1	1
43	54	F	89	Sacral	Fracture of femur neck	HTN	MSSA, Salmonella group B, Bact. thetaiotaomicron	2	Primary closure	4	36	60	46	1	0	0	0
44	55	F	88	Sacral	Stroke	HTN	Mixed flora	4	V-Y	21	83	56	28	1	0	0	0
44	56	F	89	bil. PSIS	Stroke	HTN	No growth	2	Primary closure	12	25	9	19	1	0	0	0
45	57	F	86	L't trochanter	Dementia	HTN, VHD, Atrial fibrillation	No growth	1	Primary closure	17	37	20	22	1	0	0	0
45	58	F	88	Sacral	Dementia	HTN, VHD, Atrial fibrillation	Mixed flora	2	V-Y	3	55	30	34	1	0	0	0

46	59	M	81	Sacral	Dementia	HTN, Lung cancer, COPD	Escherichia coli, Proteus mirabilis	3	V-Y	8	48	36	20	1	0	0	0
47	60	F	63	Sacral	Parkinsonism disease	Nil	Mixed flora	3	V-Y	1.5	118	110	22	1	0	0	0
48	61	M	79	R't trochanter	Dementia	HTN, CAD	Pseudo. aeruginosa	1	Primary closure	48	30	20	45	1	0	0	0
48	62	M	79	L't iliac creast	Dementia	HTN, CAD	Pseudo. aeruginosa	3	Primary closure	48	47	50	45	0	0	1	0
48	63	M	79	L't trochanter	Dementia	HTN, CAD	Pseudo. aeruginosa	2	hatchet flap	48	52	25	45	1	0	0	0
49	64	F	78	Sacral	Dementia	HTN, DM2, Cachexia	No growth	4	V-Y	26	59	64	104	1	0	0	0
49	65	F	78	Back	Dementia	HTN, DM2, Cachexia	No growth	5	rhomboid local flap	26	53	110	104	0	1	0	0
50	66	M	62	R't trochanter	Traumatic spinal cord injury	HTN, DM	Mixed flora	5	TFL hatchet flap	54	136	25	107	0	1	0	0
51	67	F	87	Sacral	Dementia	HTN	Bacteroides fragilis, Fusobacterium mortiferum	2	V-Y	23	83	72	30	1	0	0	0
52	68	F	83	L't trochanter	Stroke	HTN, DM	Peptostrepto. anaerobicus, Prevotella denticola, Bacteroides fragilis	3	Primary closure	31	61	20	55	1	0	0	0
52	69	F	83	R't trochanter	Stroke	HTN, DM	Peptostrepto. anaerobicus, Prevotella denticola, Bacteroides fragilis	2	TFL hatchet flap	31	70	40	55	1	0	0	0
52	70	F	83	Sacral	Stroke	HTN, DM	Peptostrepto. anaerobicus, Prevotella denticola, Bacteroides fragilis	1	SGAP	30	140	150	55	1	0	0	0
53	71	F	84	Sacral	Traumatic brain injury	CKD, HTN, DM2, CAD	Enterococcus raffinosus, Enterococcus faecium, Bacteroides fragilis, Peptostrepto. Magnus	2	V-Y	11	84	60	81	0	1	0	0
54	72	M	96	Sacral	Stroke	CHF, HTN, CKD	VRE	3	V-Y	3	60	96	78	0	1	0	0
55	73	M	63	L't shoulder	Stroke	HTN, DM, PAOD	Mixed flora	3	Primary closure	15	22	28	47	1	0	0	0
55	74	M	63	L't trochanter	Stroke	HTN, DM, PAOD	Mixed flora	4	Primary closure	15	36	38.5	47	1	0	0	0
55	75	M	63	Sacral	Stroke	HTN, DM, PAOD	Mixed flora	5	V-Y	15	83	80	47	1	0	0	0
56	76	M	92	Sacral	Dementia	HTN	No growth	1	V-Y	26	38	42	18	1	0	0	0

57	77	F	91	L't trochanter	Dementia	HTN	Bacteroides ovatus, Bact. distasonis	3	TFL hatchet flap	0.5	183	40	22	1	0	0	1
57	78	F	91	R't trochanter	Dementia	HTN	Bacteroides ovatus, Bact. distasonis	3	TFL hatchet flap	0.5	132	80	22	1	0	0	1
58	79	F	45	Sacral	Major trauma	Nil	MDRAB	4	Primary closure	48	70	60	51	1	0	0	0
59	80	F	85	Sacral	Dementia	HTN	Mixed flora	3	V-Y	2	146	81	43	0	1	0	0
60	81	M	89	Sacral	Fracture of femur neck	COPD, HTN	Acineto. Baumannii	2	V-Y	1.5	72	42	23	1	0	0	0
60	82	M	89	Sacral	Fracture of femur neck	COPD, HTN	Mixed flora	4	SGAP	3	181	150	35	1	0	0	0
61	83	F	85	R't trochanter	Parkinson disease	HTN, CHF	MSSA, Bacteroides fragilis	2	Primary closure	29	33	24	25	1	0	0	0
61	84	F	87	Sacral	Parkinson disease	HTN, CHF	No growth	1	Primary closure	3	21	10	11	1	0	0	0
62	85	M	63	Sacral	Stroke	HTN	No growth	1	V-Y	43	84	35	97	1	0	0	0
63	86	F	73	Sacral	Stroke	HTN, DM	Pseudo. aeruginosa	2	SGAP	42	135	144	30	1	0	0	0
64	87	M	87	Sacral	Stroke	HTN, CHF	Mixed flora	2	V-Y	38	76	72	22	1	0	0	0
65	88	M	84	Sacral	Parkinsonism	CKD, Atrial fibrillation, CAD	Pseudo. aeruginosa, Escherichia coli	2	V-Y	9	111	132	34	1	0	0	0
66	89	F	71	Sacral	Dementia	DM, dyslipidemia	Klebsiella pneumoniae	2	V-Y	10	107	42	55	1	0	0	0
67	90	F	86	Sacral	Dementia	DM	Mixed flora	3	V-Y	38	121	64	78	0	1	0	0
68	91	M	82	L't trochanter	Stroke	DM, HTN, CKD, Hyperlipidemia, Atrial fibrillation	Mixed flora	3	pALT	37	108	77	35	1	0	0	1
69	92	M	67	Sacral	CAD s/p CABG	CAD, DM, HTN, PAOD	Klebsiella pneumoniae, VRE	2	V-Y	5	86	78	280	0	0	1	1
70	93	F	84	Sacral	Dementia	CKD, CHF, HTN, Parkinson disease, Senile dementia.	Acineto. calcoaceticus-baumannii complex, Enterococcus faecium	2	V-Y	20	136	90	96	1	0	0	0
70	94	F	84	Sacral	Dementia	CKD, CHF, HTN, Parkinson disease, Senile dementia.	No growth	1	Primary closure	15	31	20	96	0	1	0	0
71	95	M	31	L't heel	Traumatic spinal cord injury	Nil	Mixed flora	2	Medial plantar	22	229	25	12	1	0	0	0

72	96	M	73	Sacral	Stroke	Nil	No growth	1	SGAP	10	100	88	14	1	0	0	0
73	97	F	58	Sacral	Breast ca with multiple mets and spinal cord compression	Breast cancer	No growth	2	V-Y	3	77	96	110	1	0	0	0
74	98	M	66	L't trochanter	ICH	Old CVA, HTN, HCV related chornic hepatitis.	Morganella morganii, Peptostrepto. Magnus, Bacteroides fragilis	4	pALT	2	173	81	57	0	1	0	0
75	99	F	70	Sacral	Dementia	HTN, DM	MRSA, Escherichia coli, Pseudo. aeruginosa	3	Primary closure	41	29	44	30	1	0	0	0
76	100	M	73	Sacral	Parkinsonism	DM	Coagulase Negative Staphylococcus	1	V-Y	1	70	20	16	1	0	0	0
77	101	M	49	Sacral	Transverse myelitis with myelopathy of lower limbs	DM, Alcoholic related liver cirrhosis	VRE	2	V-Y	2	112	60	50	1	0	0	0
78	102	M	78	R't back	*. Old CVA	HTN, DM, CAD	*. Enterococcus faecalis<br>*. Enterobacter cloacae<br>*. Klebsiella pneumoniae	1	PC+CiNPWT	38	19	12	14	1	0	0	0
79	103	M	79	Sacral	Fracture of femur, intertrochateric	Nil	Enterococcus faecium	2	SGAP	13	116	108	113	1	0	0	0
80	104	F	84	Sacral	Stroke	DM, HTN	XDRAB	3	Primary closure	4	29	36	126	0	1	0	1
81	105	F	89	Sacral	Leukoencephalopathy	DM, HTN	Mixed flora	1	V-Y	23	80	12	10	1	0	0	0
82	106	F	88	Sacral	Fracture of femur, intertrochanteric	Hypothyroidism	Escherichia coli, Morganella morganii, Bacteroides fragilis	1	V-Y	46	82	12	17	1	0	0	0
83	107	M	65	Sacral	Parkinsonism	HTN, CAD, Old CVA	Staphylococcus aureus, Corynebacterium spp	1	V-Y	8	53	35	10	1	0	0	0
84	108	F	90	Sacral	Dementia	HTN	MRSA, Morganella morganii, Escherichia coli	2	V-Y	1	65	56	28	1	0	0	1
85	109	F	71	ischial	Old fracture of femur, intertrochanteric, left	nil	No growth	3	pALT	4	213	150	38	1	0	0	0
86	110	M	65	Sacral	Traumatic spinal cord injury	Nil	Mixed flora	18	SGAP	41	93	77	Still stay due to special reason	1	1	0	0
87	111	M	77	L't ischial	Spinal cord ischemia	HTN, PAOD, Hyperuricemia	Enterococcus faecalis	1	Primary closure	55	49	18	16	1	0	0	0
88	112	F	86	Sacral	Stroke	DM, HTN, CKD, Dyslipidemia	Morganella morganii, Bacteroides fragilis, Bacteroides ovatus	2	V-Y	1	36	25	40	0	1	0	1
89	113	M	70	Sacral	Dementia	HTN, DM	Mixed flora	1	Primary closure	1.5	40	24	61	1	0	0	0
89	114	M	70	R't trochanter	Dementia	HTN, DM	Mixed flora	5	TFL hatchet flap	1.5	114	36	61	1	0	0	0

89	115	M	70	L't ischial	Dementia	HTN, DM	Mixed flora	5	Primary closure	1	40	24	61	1	0	0	0
89	116	M	70	R't ischial	Dementia	HTN, DM	Mixed flora	5	Primary closure	1	29	28	61	0	1	0	0
90	117	F	81	Sacral	Parkinsonism	VHD, CHF, HTN, ESRD, CAD, Sick sinus syndrome s/p parmanent pacemaker implantation	Bacteroides fragilis, Klebsiella pneumoniae	3	V-Y	22	79	120	43	0	1	0	0
91	118	M	80	Back	Chronic SDH	Nil	Proteus mirabilis, Enterococcus faecalis	2	Primary closure	54	57	36	32	1	0	0	0
91	119	M	80	Sacral	Chronic SDH	Nil	Proteus mirabilis, Enterococcus faecalis	3	V-Y	54	94	30	32	1	0	0	0
92	120	M	58	Sacral	Major trauma	DM, Hyperlipidemia.	No growth	2	V-Y	46	95	30	18	1	0	0	0
93	121	F	70	Sacral	Spondylolisthesis	CHF	MRSA	2	V-Y	31	130	132	97	0	1	0	0
94	122	F	59	Sacral	Parkinsonism	DM	Mixed flora	4	Primary closure	5	38	40	86	1	0	0	0
94	123	F	59	R't trochanter	Parkinsonism	DM	Mixed flora	7	pALT	4.5	183	70	86	1	0	0	0
94	124	F	59	L't trochanter	Parkinsonism	DM	Mixed flora	9	pALT	4	146	117	86	1	0	0	0
95	125	F	62	Sacral	CNS lymphoma	HTN	Enterococcus faecalis	2	V-Y	54	49	20	34	0	1	0	0
96	126	F	87	Sacral	Fracture of femur	ESRD, VHD, CHF, Atrial fibrillation	MRSA, Escherichia coli, Klebsiella pneumoniae	1	V-Y	2	50	49	439	1	0	0	0
97	127	F	73	Sacral	Parkinsonism	HTN, DM, CAD	Mixed flora	3	V-Y	50	151	150	40	1	0	0	0
98	128	M	86	Sacral	Stroke	HTN	Enterococcus faecalis, Proteus mirabilis, Pseudo. aeruginosa,	4	V-Y	19	108	144	45	1	0	0	0
99	129	M	74	R't ischial	Poliomyelitis with paralysis of lower limbs	Nil	No growth	1	Primary closure	16	23	20	17	1	0	0	0
100	130	M	89	Sacral	Major Depression Disease	Nil	Klebsiella pneumoniae, Pseudo. aeruginosa, Enterococcus faecalis	3	Primary closure	1	65	130	33	1	0	0	0
101	131	F	88	Sacral	Dementia	Nil	Bacteroides fragilis, Peptostrepto. asaccharolyticus, Viridans streptococcus gr, Enterococcus faecalis	3	V-Y	1	106	36	26	1	0	0	0
102	132	F	75	Sacral	Dementia	Nil	Mixed flora	2	SGAP	13	150	72	58	0	1	0	0

103	133	M	21	Sacral	Cervical spine injury	Nil	No growth	2	V-Y	27	59	15	339	1	0	0	0
103	134	M	23	R't ischial	Cervical spine injury	Nil	No growth	2	pALT	3	229	50	25	1	0	0	0
104	135	M	73	Sacral	Dementia	HTN, DM	Enterococcus faecalis, Proteus mirabilis, Peptostrepto. anaerobicus, Prevotella spp.	5	SGAP	1	110	126	56	0	1	0	0
105	136	M	50	Sacral	Fracture of femur	HTN, DM	Mixed flora	6	V-Y	54	68	56	109	0	0	1	0
105	137	M	50	Sacral	Fracture of femur	HTN, DM	Mixed flora	6	2nd V-Y	54	81	48	109	1	0	0	0
106	138	M	69	Sacral	Stroke	DM, CKD	Enterococcus faecium, Enterococcus faecalis	2	V-Y	4.5	39	25	153	0	1	0	0
107	139	M	68	Sacral	Traumatic spinal cord injury	DM	Pseudo. aeruginosa, Proteus mirabilis	2	V-Y	1	53	72	20	1	0	0	0
108	140	M	66	Sacral	Fracture of femur	Nil	Mixed flora	4	V-Y	4	87	400	114	0	1	0	0
109	141	M	20	Sacral	Traumatic spinal cord injury	Nil	Coagulase Negative Staphylococcus	2	SGAP	6	117	25	41	0	1	0	1
110	142	F	87	Sacral	Dementia	ESRD, DM	Pseudo. aeruginosa, Enterococcus faecium	14	V-Y	11	83	56	80	0	0	1	0
110	143	F	87	Sacral	Dementia	ESRD, DM	Pseudo. aeruginosa, Enterococcus faecium	2	2nd V-Y	11	81	48	80	0	1	0	0
111	144	F	53	Sacral	Schizophrenia	Nil	No growth	2	V-Y	1	68	25	32	1	0	0	0
112	145	F	77	Sacral	Dementia	Nil	Mixed flora	2	V-Y	1	54	36	19	1	0	0	0
113	146	M	60	Sacral	Amphetamina withdraw, Major depression	Nil	MRSA	1	V-Y	2	73	35	28	1	0	0	0
114	147	M	84	Sacral	Dementia	HTN, CKD, Dementia	Proteus mirabilis, Flavobacterium species, Corynebacterium spp.	5	SGAP	7	194	108	246	0	1	0	0
115	148	M	91	Sacral	Dementia	HTN	Enterococcus faecalis, Pseudo. Aeruginosa, Peptostrepto. Magnus	1	V-Y	54	45	48	10	1	0	0	0
116	149	F	85	R't trochanter	Fracture of femur	HTN, VHD, DM2	No growth	1	TFL hatchet flap	52	38	20	15	1	0	0	0
117	150	F	74	Sacral	Dementia	DM	Mixed flora	1	Primary closure	2	43	40	27	1	0	0	0
118	151	F	92	Sacral	Stroke	HTN, DM	No growth	1	V-Y	56	99	30	22	1	0	0	0

119	152	F	76	Sacral	Status epilepticus	HTN	VRE	2	V-Y	46	50	30	48	1	0	0	0	
120	153	F	76	Sacral	Dementia	Nil	No growth	2	V-Y	1	46	45	31	0	1	0	0	
121	154	F	81	Sacral	Peritoneal carcinomatosis	HTN, Atrial fibrillation	Enterococcus faecium, Klebsiella pneumoniae	3	SGAP	5	93	110	83	0	1	0	0	
122	155	F	83	Sacral	Parkinson disease	Nil	Mixed flora	2	V-Y	37	55	12	21	1	0	0	0	
123	156	M	84	Sacral	Parkinson's disease	HTN	Mixed flora	3	V-Y	4	119	180	65	0	1	0	0	
124	157	F	59	Sacral	Fracture of femur	DM, Schizophrenia	Pseudo. aeruginosa, Proteus mirabilis	2	V-Y	25	74	64	28	1	0	0	0	
125	158	M	87	Sacral	Alzheimer's disease	HTN, DM2, Prostate ca	Pseudo. aeruginosa	1	V-Y	26	66	9	16	1	0	0	0	
126	159	M	85	R't trochanter	Spondylopathy	HTN	No growth	2	GM V-Y	48	38	12	10	1	0	0	0	
126	160	M	85	Sacral	Recurrent perineal abscess with secondary infection	HTN	No growth	2	V-Y	18	63	42	32	1	0	0	0	
127	161	F	81	Sacral	*. Old CVA	HTN, DM, CAD	*. Escherichia coli	2	PC+CiNPWT	17	12	10	10	1	0	0	0	
128	162	F	92	Sacral	Dementia	Nil	Enterococcus faecalis, Escherichia coli	2	SGAP	23	130	72	23	1	0	0	0	
